# Supplementary material for: Mechanisms and drivers of belemnite body-size dynamics across the Pliensbachian–Toarcian crisis
Source: R Soc Open Sci. 2019 Dec 11;6(12):190494. doi: 10.1098/rsos.190494 (PMC6936285; doi:10.1098/rsos.190494)
Supplement: Supplementary figures [file rsos190494supp1.docx]

Supplementary figures


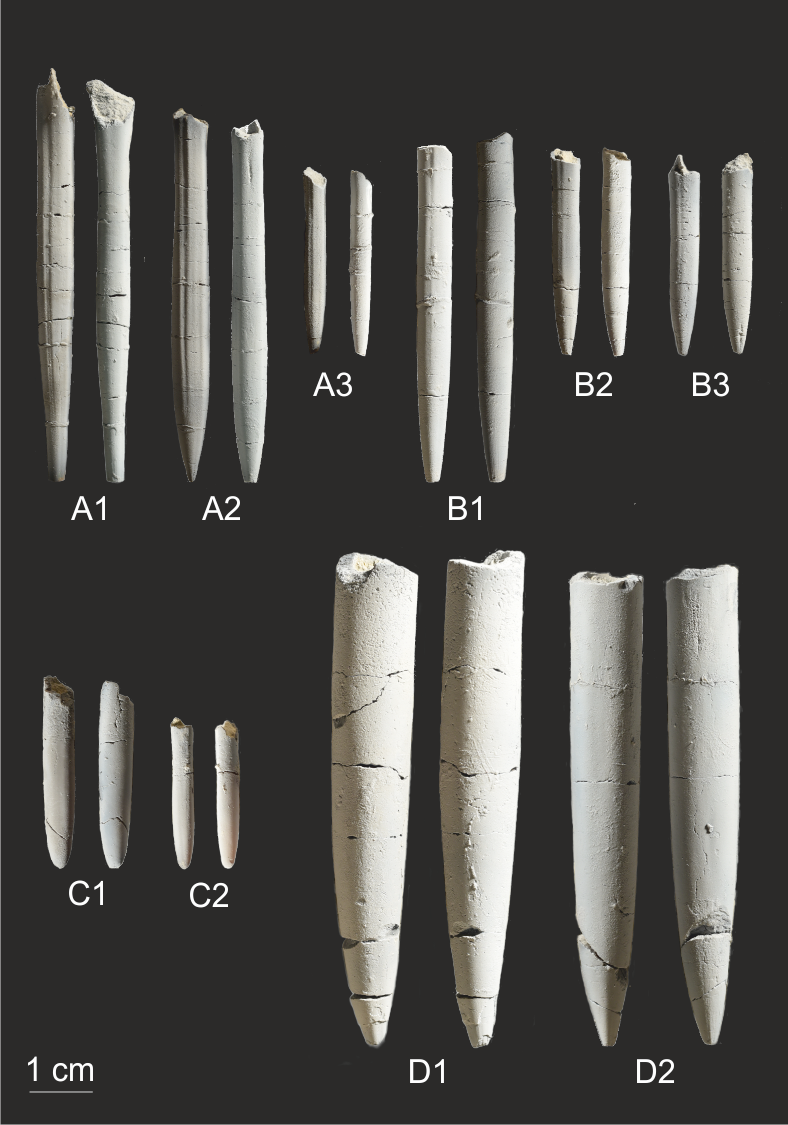


*Figure S1 – Selected belemnites from the Upper Pliensbachian-Lower Toarcian of Peniche: A1 - Bairstowius sp. A (adult from bed P1); A2- Bairstowius sp. A (adult from bed P2); A3- Bairstowius sp. A (juvenile from bed P1); B1- Pseudohastites longiformis (adult from bed P4); B2 and B3 - Pseudohastites longiformis (juveniles from bed P4); C1 and C2- Hastitidae. sp. indet. (from bed P5); D1 and D2 - Passaloteuthis milleri (adults from bed P2).*

*The left side corresponds to lateral view and right side corresponds to dorsal/ventral view.*


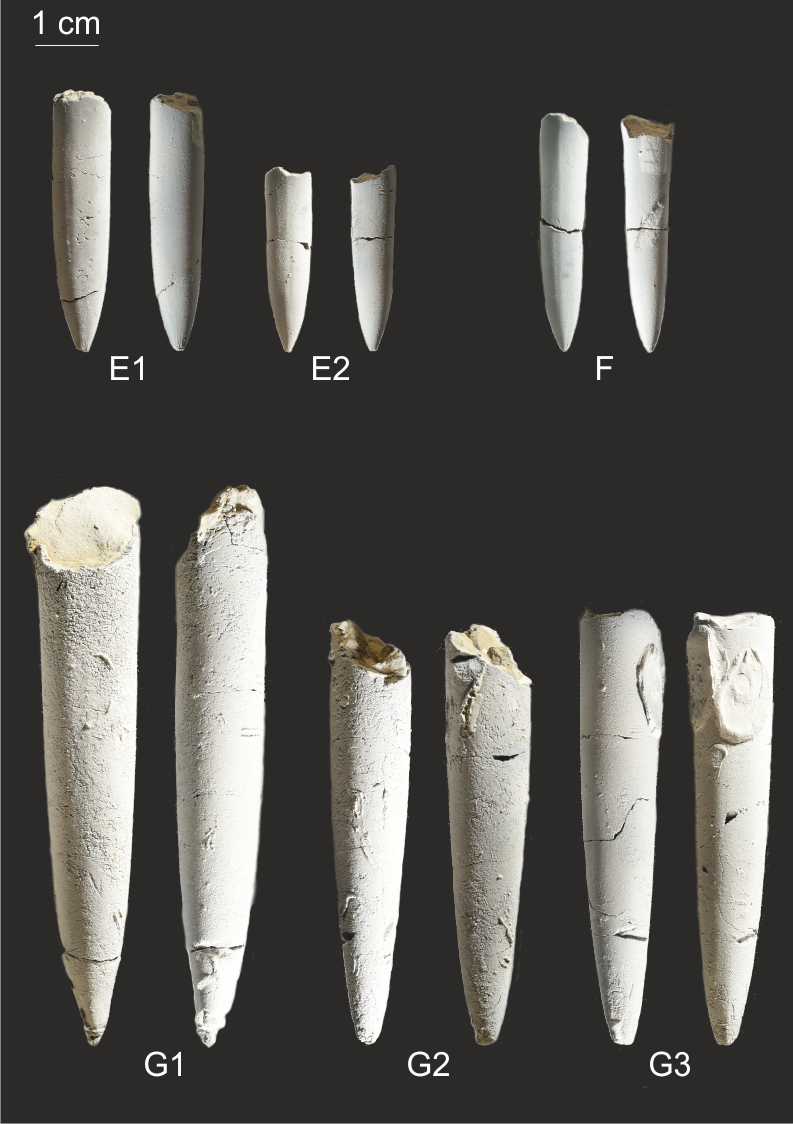


*Figure S2 – Selected belemnites from the Upper Pliensbachian-Lower Toarcian of Peniche: E1- Parapassaloteuthis sp. A (adult from bed P3); E2 - Parapassaloteuthis sp. A (juvenile from bed P3); F - Passaloteuthis sp. juv. (from bed P1); G1-G2 - Passaloteuthis bisulcata (adults from bed P5); G3 - Passaloteuthis bisulcata (adult from bed P2). The left side corresponds to lateral view and right side corresponds to dorsal/ventral view.*


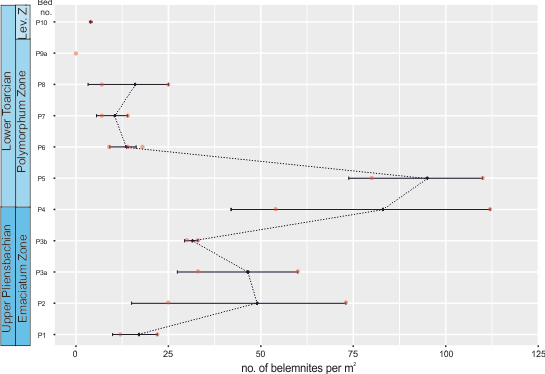


Figure S3 – Belemnite absolute abundance (no. of belemnites collected per m^2^). The interrupted black line connects the mean (black dots) for the beds where more than one quadrat were analysed. Each red dot represents a quadrat of 1 m^2^. The error bars represent the standard deviation of the mean.


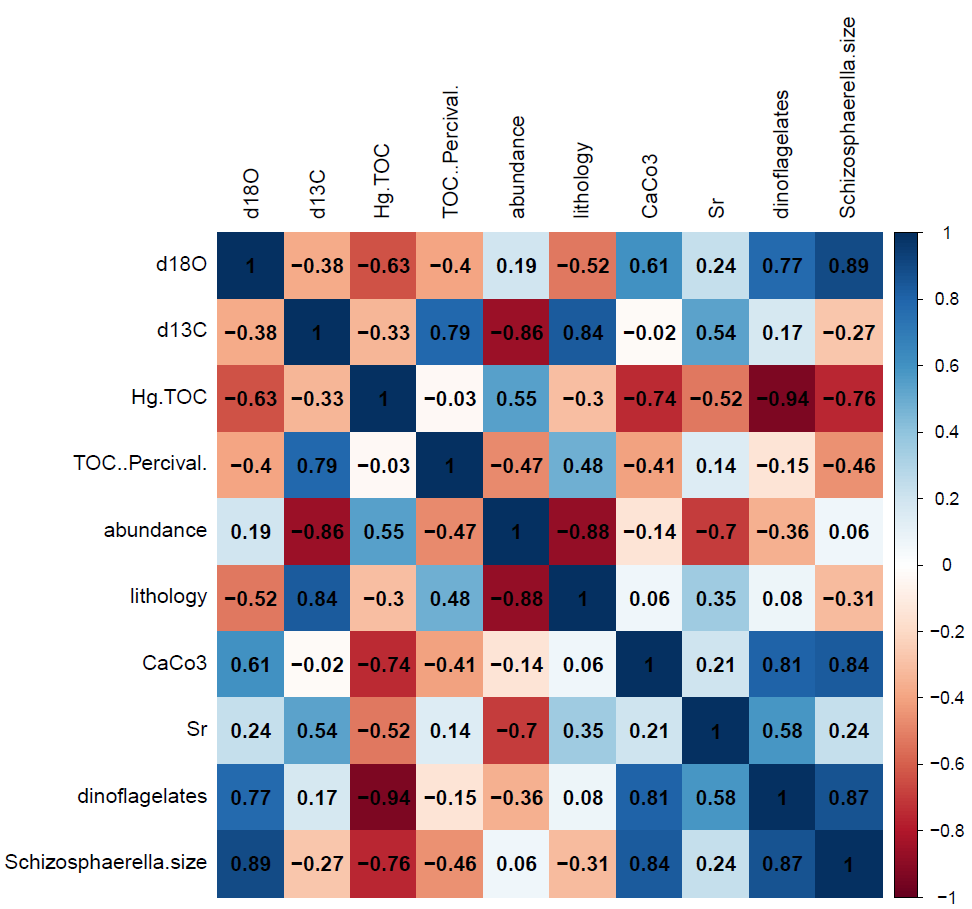


*Figure S4 – Correlation matrix depicting the relation between the different environmental variables available in the literature for the Peniche section. Note that only δ^18^O, δ^13^C, Hg.TOC, lithology and abundance were included as explanatory variables in the regression analysis due to the high collinearity among the remaining ones. See TS1 for details.*


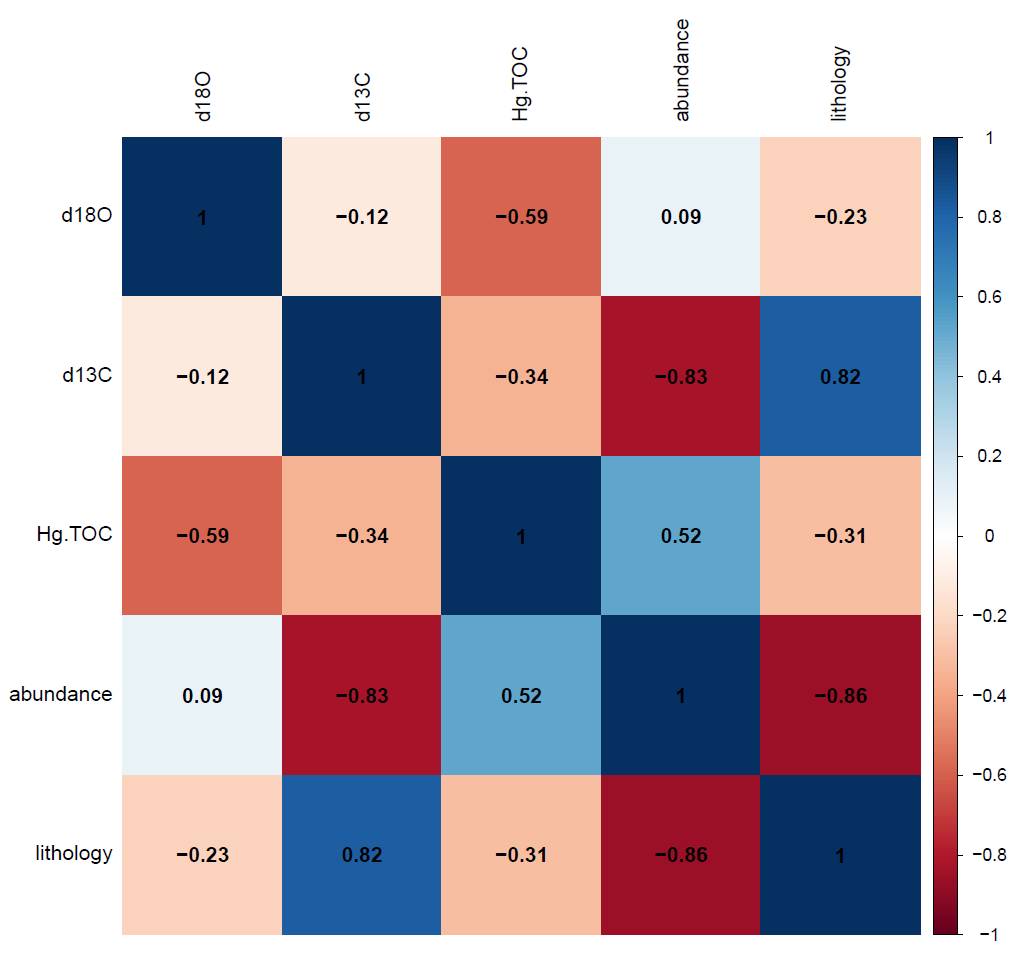


*Figure S5– Correlation matrix depicting the relation between the different explanatory variables used in the performed regression analysis between belemnite body-size and abiotic parameters.*

*
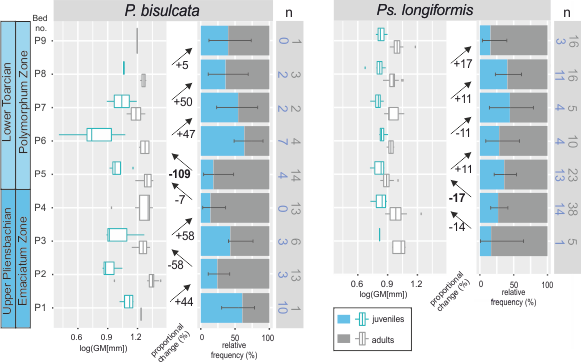
Figure S6 – Belemnite body–size variation (GM), proportional body-size change, relative frequency of ontogenetic stages and sample size (n) across the Upper Pliensbachian-Lower Toarcian of Peniche at species scale (P. bisulcata and Ps. longiformis). Passaloteuthis genus was used to calculate the relative frequency of ontogenetic stages of P. bisulcata due to the difficulty of a species level classification of the juvenile specimens of Passaloteuthis genus. The error bars correspond to the 95 % confidence interval. Note that the sample size (n) corresponds to species level.*


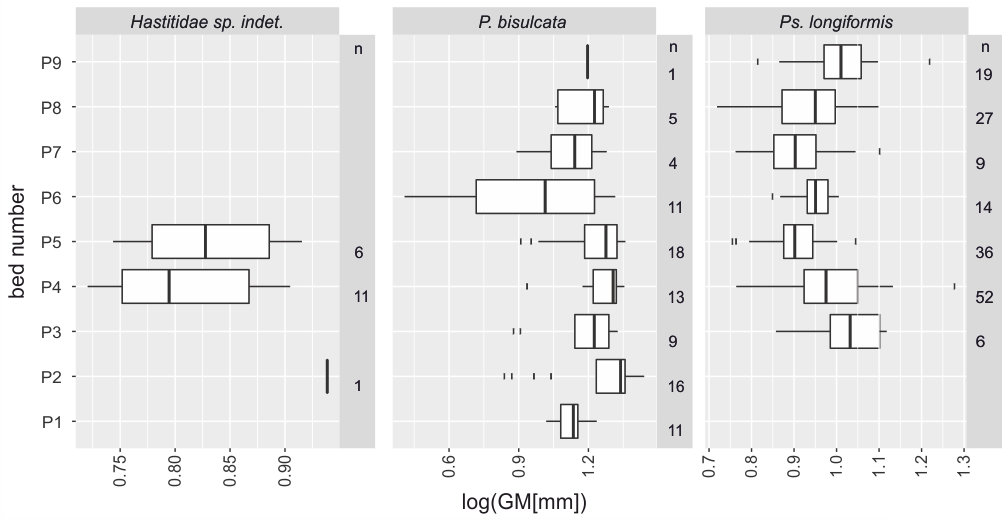


*Figure S7 – Body–size variation (GM) and sample size (n) of Pliensbachian-Toarcian boundary-crossers (P. bisulcata, Ps. longiformis and Hastitidae sp. indet.) of the Peniche section. Although Parapassaloteuthis sp. A also crosses the Pli-Toa boundary, we have no complete specimens across this interval and therefore, no accurate estimate of body–size.*


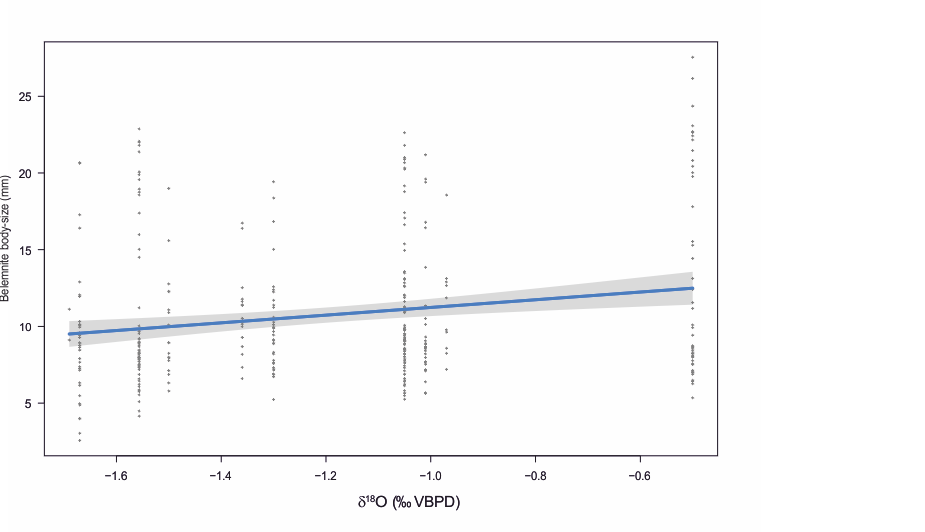


Figure S8 – Relationship between the seawater palaeotemperature proxy (δ^18^O) and belemnite body–size (GM) during the Upper Pliensbachian-Lower Toarcian of Peniche. The grey area corresponds to the 95 % confidence interval.


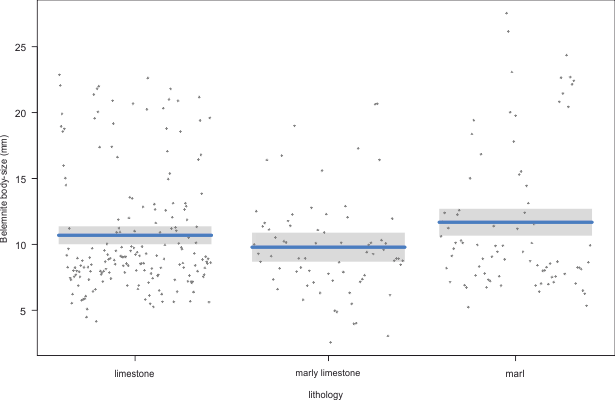


Figure S9 – Relationship between belemnite body–size (GM) and lithology for the studied interval in Peniche. Adjusted R^2^=–0.0009292; p–value= 0.4083. The grey area corresponds to the 95 % confidence interval.


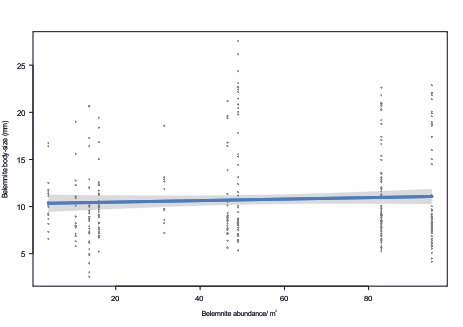


Figure S10 – Relationship between belemnite body–size (GM) and belemnite absolute abundance (no. of belemnites/ m^2^) for the studied interval in Peniche. The grey area corresponds to the 95 % confidence interval. Adjusted R^2^=9.356e–05; p–value=0.3105.
