# Supplementary material for: Mechanisms and drivers of belemnite body-size dynamics across the Pliensbachian–Toarcian crisis
Source: R Soc Open Sci. 2019 Dec 11;6(12):190494. doi: 10.1098/rsos.190494 (PMC6936285; doi:10.1098/rsos.190494)
Supplement: R script [file rsos190494supp3.docx]

R script for testing the hypotheses of a relationship between belemnites body-size and environmental perturbations and sedimentary properties

Supplementary material of Rita et al. (“Mechanisms and drivers of belemnite body size dynamics across the Pliensbachian-Toarcian crisis”)

1. ***GLS models (assemblage scale)***

############### Multiple linear regression with GLS method ###############################

library(nlme)

library(qpcR)

library(corrplot)

############ dealing with NA's

GMdat<-dat3[is.na(dat3$GM.1)==F,]# removing NA from GM column

###############examining bivariate relationships between the variables

GMdat<-GMdat[rowSums(is.na(GMdat[,c("GM.1","d18O","Hg.TOC","d13C")]))==0,]#excludes NA

GMdat<-select(GMdat, GM.1, d18O, d13C, Hg.TOC)#select the variables we will use in the models

cor(GMdat, use="pairwise.complete.obs") #plots correlation matrix

newdatacor = cor(GMdat[1:10])#choose the varables to include in the matrix

corrplot(newdatacor, method = "number", bg="grey", addgrid.col="black", tl.col="black")#plots correlation matrix with colours

################### fit a model removing properly the NA's from all the variables at the same time, **without removing the effects of lithology and abundance of belemnites**

gls.null <- gls(GM.1~1, correlation=corARMA(p=1), method="ML",data=GMdat[rowSums(is.na(GMdat[,c("GM.1","d18O","d13C", "Hg.TOC")]))==0,])

gls.1 <- gls(GM.1~d18O+d13C+Hg.TOC, correlation=corARMA(p=1), method="ML",data=GMdat[rowSums(is.na(GMdat[,c("GM.1","d18O","d13C", "Hg.TOC")]))==0,])

gls.2 <- gls(GM.1~d18O+d13C, correlation=corARMA(p=1), method="ML",data=GMdat[rowSums(is.na(GMdat[,c("GM.1","d18O","d13C", "Hg.TOC")]))==0,])

gls.3 <- gls(GM.1~Hg.TOC+d13C, correlation=corARMA(p=1), method="ML",data=GMdat[rowSums(is.na(GMdat[,c("GM.1","d18O","d13C", "Hg.TOC")]))==0,])

gls.4 <- gls(GM.1~Hg.TOC+d18O, correlation=corARMA(p=1), method="ML",data=GMdat[rowSums(is.na(GMdat[,c("GM.1","d18O","d13C", "Hg.TOC")]))==0,])

gls.5 <- gls(GM.1~d18O, correlation=corARMA(p=1), method="ML",data=GMdat[rowSums(is.na(GMdat[,c("GM.1","d18O","d13C", "Hg.TOC")]))==0,])

gls.6 <- gls(GM.1~Hg.TOC, correlation=corARMA(p=1), method="ML",data=GMdat[rowSums(is.na(GMdat[,c("GM.1","d18O","d13C", "Hg.TOC")]))==0,])

gls.7 <- gls(GM.1~d13C, correlation=corARMA(p=1), method="ML",data=GMdat[rowSums(is.na(GMdat[,c("GM.1","d18O","d13C", "Hg.TOC")]))==0,])

#Computing and comparing the weightings and AICc values for all models in order to check which of the models is the best

weighted<-function (aic){

aic.wt <- exp(-0.5 * (aic - min(aic)))/sum(exp(-0.5 * (aic - min(aic))))

return(aic.wt)

}

weighted(c(AICc(gls.null), AICc(gls.1), AICc(gls.2), AICc(gls.3), AICc(gls.4),AICc(gls.5), AICc(gls.6), AICc(gls.7)))

AICcscores <- (c(AICc(gls.null), AICc(gls.1), AICc(gls.2), AICc(gls.3), AICc(gls.4), AICc(gls.5), AICc(gls.6), AICc(gls.7)))

AICcscores #Display the AICc scores

# Deriving a global p-value using ANOVA for the model no. 5, the best one for the assemblage scale:

anova(gls.null, gls.5)

################### fit a model removing properly the NA's from all the variables at the same time, **correcting for the effects of lithology and abundance of belemnites**

#Dealing with NA’s

GMdat<-GMdat[rowSums(is.na(GMdat[,c("GM.1","d18O","d13C","abundance","lithology","Hg.TOC")]))==0,]

#Fit a linear model between body-size (GM.1) and lithology and abundance

model<-lm(GMdat$GM.1~GMdat$abundance+GMdat$lithology)

#Save the residuals of the linear model as a variable

GMdat$GMresiduals<-model$resid

#Fit GLS models using the residuals of the previous linear regression

gls.null <- gls(GMresiduals~1, correlation=corARMA(p=1), method="ML",data=GMdat[rowSums(is.na(GMdat[,c("GMresiduals","d18O","d13C", "Hg.TOC")]))==0,])

gls.1 <- gls(GMresiduals~d18O+d13C+Hg.TOC, correlation=corARMA(p=1), method="ML",data=GMdat[rowSums(is.na(GMdat[,c("GMresiduals","d18O","d13C", "Hg.TOC")]))==0,])

gls.2 <- gls(GMresiduals~d18O+d13C, correlation=corARMA(p=1), method="ML",data=GMdat[rowSums(is.na(GMdat[,c("GMresiduals","d18O","d13C", "Hg.TOC")]))==0,])

gls.3 <- gls(GMresiduals~Hg.TOC+d13C, correlation=corARMA(p=1), method="ML",data=GMdat[rowSums(is.na(GMdat[,c("GMresiduals","d18O","d13C", "Hg.TOC")]))==0,])

gls.4 <- gls(GMresiduals~Hg.TOC+d18O, correlation=corARMA(p=1), method="ML",data=GMdat[rowSums(is.na(GMdat[,c("GMresiduals","d18O","d13C", "Hg.TOC")]))==0,])

gls.5 <- gls(GMresiduals~d18O, correlation=corARMA(p=1), method="ML",data=GMdat[rowSums(is.na(GMdat[,c("GMresiduals","d18O","d13C", "Hg.TOC")]))==0,])

gls.6 <- gls(GMresiduals~Hg.TOC, correlation=corARMA(p=1), method="ML",data=GMdat[rowSums(is.na(GMdat[,c("GMresiduals","d18O","d13C", "Hg.TOC")]))==0,])

gls.7 <- gls(GMresiduals~d13C, correlation=corARMA(p=1), method="ML",data=GMdat[rowSums(is.na(GMdat[,c("GMresiduals","d18O","d13C", "Hg.TOC")]))==0,])

# Computing and comparing the weightings and AICc values for all models in order to check which of the models is the best

weighted<-function (aic){

aic.wt <- exp(-0.5 * (aic - min(aic)))/sum(exp(-0.5 * (aic - min(aic))))

return(aic.wt)

}

weighted(c(AICc(gls.null), AICc(gls.1), AICc(gls.2), AICc(gls.3), AICc(gls.4),AICc(gls.5), AICc(gls.6), AICc(gls.7)))

AICcscores <- (c(AICc(gls.null), AICc(gls.1), AICc(gls.2), AICc(gls.3), AICc(gls.4), AICc(gls.5), AICc(gls.6), AICc(gls.7)))

AICcscores #Display AICc scores

anova(gls.null, gls.5) #calculating the p-value of model no. 5, the best one according to the AICc scores at the assemblage scale, after correcting for the effects of abundance and lithology

1. ***Rego et al. method***

(this should be complemented with Table S3)

##### beds P925-P949 (=P1-P2)

dat1$Rego[dat1$Groups=="Bairstowius sp. A" & dat1$New.level=="P925"] <-"pre-survivor"

dat1$Rego[dat1$Groups=="P. bisulcata" & dat1$New.level=="P925"] <-"pre-survivor"

dat1$Rego[dat1$Groups=="P. milleri" & dat1$New.level=="P925"] <-"pre-survivor"

#dat1$Rego[dat1$Groups=="indeterminable" & dat1$New.level=="P925"] <-"survivor"

presurvivors <- subset(dat1, Rego == "pre-survivor")

presurvivorsGM <- ddply(presurvivors, .(New.level), summarise, med = median(GM.1, na.rm=TRUE),freq=length(GM.1))

dat1$Rego[dat1$Groups=="Bairstowius sp. A" & dat1$New.level=="P949"] <-"pos-survivor"

dat1$Rego[dat1$Groups=="P. bisulcata" & dat1$New.level=="P949"] <-"pos-survivor"

dat1$Rego[dat1$Groups=="P. milleri" & dat1$New.level=="P949"] <-"pos-survivor"

#dat1$Rego[dat1$Groups=="indeterminable" & dat1$New.level=="P949"] <-"survivor"

possurvivors <- subset(dat1, Rego == "pos-survivor")

possurvivorsL <- ddply(possurvivors, .(New.level), summarise, med = median(GM.1, na.rm=TRUE),freq=length(GM.1))

#####beds P949-P961 (=P2-P3)

dat1$Rego[dat1$Groups=="Bairstowius sp. A" & dat1$New.level=="P949"] <-"pre-survivor"

dat1$Rego[dat1$Groups=="P. bisulcata" & dat1$New.level=="P949"] <-"pre-survivor"

dat1$Rego[dat1$Groups=="P. milleri" & dat1$New.level=="P949"] <-"pre-survivor"

#dat1$Rego[dat1$Groups=="indeterminable" & dat1$New.level=="P949"] <-"pre-survivor"

#dat1$Rego[dat1$Groups=="Hastitidae sp. indet." & dat1$New.level=="P949"] <-"pre-survivor"

presurvivors <- subset(dat1, Rego == "pre-survivor")

presurvivorsL <- ddply(presurvivors, .(New.level), summarise, med = median(GM.1, na.rm=TRUE),freq=length(GM.1))

dat1$Rego[dat1$Groups=="Bairstowius sp. A" & dat1$New.level=="P961"] <-"pos-survivor"

dat1$Rego[dat1$Groups=="P. bisulcata" & dat1$New.level=="P961"] <-"pos-survivor"

dat1$Rego[dat1$Groups=="P. milleri" & dat1$New.level=="P961"] <-"pos-survivor"

#dat1$Rego[dat1$Groups=="indeterminable" & dat1$New.level=="P961"] <-"pos-survivor"

#dat1$Rego[dat1$Groups=="Hastitidae sp. indet." & dat1$New.level=="P961"] <-"pos-survivor"

possurvivors <- subset(dat1, Rego == "pos-survivor")

possurvivorsL <- ddply(possurvivors, .(New.level), summarise, med = median(GM.1, na.rm=TRUE),freq=length(GM.1))

#####beds P961-P982 (=P3-P4)

dat1$Rego[dat1$Groups=="Parap. sp. 1" & dat1$New.level=="P961"] <-"pre-survivor"

dat1$Rego[dat1$Groups=="P. bisulcata" & dat1$New.level=="P961"] <-"pre-survivor"

dat1$Rego[dat1$Groups=="P. milleri" & dat1$New.level=="P961"] <-"pre-survivor"

#dat1$Rego[dat1$Groups=="indeterminable" & dat1$New.level=="P961"] <-"pre-survivor"

#dat1$Rego[dat1$Groups=="Hastitidae sp. indet." & dat1$New.level=="P961"] <-"pre-survivor"

dat1$Rego[dat1$Groups=="P. cf. longiformis" & dat1$New.level=="P961"] <-"pre-survivor"

presurvivors <- subset(dat1, Rego == "pre-survivor")

presurvivorsL <- ddply(presurvivors, .(New.level), summarise, med = median(GM.1, na.rm=TRUE),freq=length(GM.1))

dat1$Rego[dat1$Groups=="Parap. sp. 1" & dat1$New.level=="P982"] <-"pos-survivor"

dat1$Rego[dat1$Groups=="P. bisulcata" & dat1$New.level=="P982"] <-"pos-survivor"

dat1$Rego[dat1$Groups=="P. milleri" & dat1$New.level=="P982"] <-"pos-survivor"

#dat1$Rego[dat1$Groups=="indeterminable" & dat1$New.level=="P982"] <-"pos-survivor"

#dat1$Rego[dat1$Groups=="Hastitidae sp. indet." & dat1$New.level=="P982"] <-"pos-survivor"

dat1$Rego[dat1$Groups=="P. cf. longiformis" & dat1$New.level=="P982"] <-"pos-survivor"

possurvivors <- subset(dat1, Rego == "pos-survivor")

possurvivorsL <- ddply(possurvivors, .(New.level), summarise, med = median(GM.1, na.rm=TRUE),freq=length(GM.1))

##### beds P982-P984 (=P4-P5)

#dat1$Rego[dat1$Groups=="Parap. sp. 1" & dat1$New.level=="P982"] <-"pre-survivor"

dat1$Rego[dat1$Groups=="P. bisulcata" & dat1$New.level=="P982"] <-"pre-survivor"

dat1$Rego[dat1$Groups=="indeterminable" & dat1$New.level=="P982"] <-"pre-survivor"

dat1$Rego[dat1$Groups=="Hastitidae sp. indet." & dat1$New.level=="P982"] <-"pre-survivor"

dat1$Rego[dat1$Groups=="P. cf. longiformis" & dat1$New.level=="P982"] <-"pre-survivor"

presurvivors <- subset(dat1, Rego == "pre-survivor")

presurvivorsL <- ddply(presurvivors, .(New.level), summarise, med = median(GM.1, na.rm=TRUE),freq=length(GM.1))

#dat1$Rego[dat1$Groups=="Parap. sp. 1" & dat1$New.level=="P984"] <-"pos-survivor"

dat1$Rego[dat1$Groups=="P. bisulcata" & dat1$New.level=="P984"] <-"pos-survivor"

dat1$Rego[dat1$Groups=="indeterminable" & dat1$New.level=="P984"] <-"pos-survivor"

dat1$Rego[dat1$Groups=="Hastitidae sp. indet." & dat1$New.level=="P984"] <-"pos-survivor"

dat1$Rego[dat1$Groups=="P. cf. longiformis" & dat1$New.level=="P984"] <-"pos-survivor"

possurvivors <- subset(dat1, Rego == "pos-survivor")

possurvivorsL <- ddply(possurvivors, .(New.level), summarise, med = median(GM.1, na.rm=TRUE),freq=length(GM.1))

#####beds P984-P8 (=P5-P6)

#dat1$Rego[dat1$Groups=="Parap. sp. 1" & dat1$New.level=="P984"] <-"pre-survivor"

dat1$Rego[dat1$Groups=="P. bisulcata" & dat1$New.level=="P984"] <-"pre-survivor"

#dat1$Rego[dat1$Groups=="indeterminable" & dat1$New.level=="P984"] <-"pre-survivor"

#dat1$Rego[dat1$Groups=="Passaloteuthis sp. juv" & dat1$New.level=="P984"] <-"pre-survivor"

dat1$Rego[dat1$Groups=="P. cf. longiformis" & dat1$New.level=="P984"] <-"pre-survivor"

presurvivors <- subset(dat1, Rego == "pre-survivor")

presurvivorsL <- ddply(presurvivors, .(New.level), summarise, med = median(GM.1, na.rm=TRUE),freq=length(GM.1))

#dat1$Rego[dat1$Groups=="Parap. sp. 1" & dat1$New.level=="P8"] <-"pos-survivor"

dat1$Rego[dat1$Groups=="P. bisulcata" & dat1$New.level=="P8"] <-"pos-survivor"

#dat1$Rego[dat1$Groups=="indeterminable" & dat1$New.level=="P8"] <-"pos-survivor"

#dat1$Rego[dat1$Groups=="Passaloteuthis sp. juv" & dat1$New.level=="P8"] <-"pos-survivor"

dat1$Rego[dat1$Groups=="P. cf. longiformis" & dat1$New.level=="P8"] <-"pos-survivor"

possurvivors <- subset(dat1, Rego == "pos-survivor")

possurvivorsL <- ddply(possurvivors, .(New.level), summarise, med = median(GM.1, na.rm=TRUE),freq=length(GM.1))

#####beds P8-P12 (=P6-P7)

dat1$Rego[dat1$Groups=="Parap. sp. 1" & dat1$New.level=="P8"] <-"pre-survivor"

dat1$Rego[dat1$Groups=="P. bisulcata" & dat1$New.level=="P8"] <-"pre-survivor"

#dat1$Rego[dat1$Groups=="indeterminable" & dat1$New.level=="P8"] <-"pre-survivor"

dat1$Rego[dat1$Groups=="Passaloteuthis sp. juv" & dat1$New.level=="P8"] <-"pre-survivor"

dat1$Rego[dat1$Groups=="P. cf. longiformis" & dat1$New.level=="P8"] <-"pre-survivor"

#dat1$Rego[dat1$Groups=="P. milleri" & dat1$New.level=="P8"] <-"survivor"

presurvivors <- subset(dat1, Rego == "pre-survivor")

presurvivorsL <- ddply(presurvivors, .(New.level), summarise, med = median(GM.1, na.rm=TRUE),freq=length(GM.1))

dat1$Rego[dat1$Groups=="Parap. sp. 1" & dat1$New.level=="P12"] <-"pos-survivor"

dat1$Rego[dat1$Groups=="P. bisulcata" & dat1$New.level=="P12"] <-"pos-survivor"

#dat1$Rego[dat1$Groups=="indeterminable" & dat1$New.level=="P12"] <-"pos-survivor"

dat1$Rego[dat1$Groups=="Passaloteuthis sp. juv" & dat1$New.level=="P12"] <-"pos-survivor"

dat1$Rego[dat1$Groups=="P. cf. longiformis" & dat1$New.level=="P12"] <-"pos-survivor"

#dat1$Rego[dat1$Groups=="P. milleri" & dat1$New.level=="P12"] <-"survivor"

possurvivors <- subset(dat1, Rego == "pos-survivor")

possurvivorsL <- ddply(possurvivors, .(New.level), summarise, med = median(GM.1, na.rm=TRUE),freq=length(GM.1))

#####beds P12-P14 (=P7-P8)

dat1$Rego[dat1$Groups=="Parap. sp. 1" & dat1$New.level=="P12"] <-"pre-survivor"

dat1$Rego[dat1$Groups=="P. bisulcata" & dat1$New.level=="P12"] <-"pre-survivor"

#dat1$Rego[dat1$Groups=="indeterminable" & dat1$New.level=="P12"] <-"pre-survivor"

dat1$Rego[dat1$Groups=="Passaloteuthis sp. juv" & dat1$New.level=="P12"] <-"pre-survivor"

dat1$Rego[dat1$Groups=="P. cf. longiformis" & dat1$New.level=="P12"] <-"pre-survivor"

dat1$Rego[dat1$Groups=="P. milleri" & dat1$New.level=="P12"] <-"survivor"

presurvivors <- subset(dat1, Rego == "pre-survivor")

presurvivorsL <- ddply(presurvivors, .(New.level), summarise, med = median(GM.1, na.rm=TRUE),freq=length(GM.1))

dat1$Rego[dat1$Groups=="Parap. sp. 1" & dat1$New.level=="P14"] <-"pos-survivor"

dat1$Rego[dat1$Groups=="P. bisulcata" & dat1$New.level=="P14"] <-"pos-survivor"

#dat1$Rego[dat1$Groups=="indeterminable" & dat1$New.level=="P14"] <-"pos-survivor"

dat1$Rego[dat1$Groups=="Passaloteuthis sp. juv" & dat1$New.level=="P14"] <-"pos-survivor"

dat1$Rego[dat1$Groups=="P. cf. longiformis" & dat1$New.level=="P14"] <-"pos-survivor"

dat1$Rego[dat1$Groups=="P. milleri" & dat1$New.level=="P14"] <-"pos-survivor"

possurvivors <- subset(dat1, Rego == "pos-survivor")

possurvivorsL <- ddply(possurvivors, .(New.level), summarise, med = median(GM.1, na.rm=TRUE),freq=length(GM.1))

#####beds P14-P20 (=P8-P9)

dat1$Rego[dat1$Groups=="Parap. sp. 1" & dat1$New.level=="P14"] <-"pre-survivor"

dat1$Rego[dat1$Groups=="P. bisulcata" & dat1$New.level=="P14"] <-"pre-survivor"

#dat1$Rego[dat1$Groups=="indeterminable" & dat1$New.level=="P14"] <-"pre-survivor"

#dat1$Rego[dat1$Groups=="Passaloteuthis sp. juv" & dat1$New.level=="P14"] <-"pre-survivor"

dat1$Rego[dat1$Groups=="P. cf. longiformis" & dat1$New.level=="P14"] <-"pre-survivor"

dat1$Rego[dat1$Groups=="P. milleri" & dat1$New.level=="P14"] <-"survivor"

presurvivors <- subset(dat1, Rego == "pre-survivor")

presurvivorsL <- ddply(presurvivors, .(New.level), summarise, med = median(GM.1, na.rm=TRUE),freq=length(GM.1))

dat1$Rego[dat1$Groups=="Parap. sp. 1" & dat1$New.level=="P20"] <-"pos-survivor"

dat1$Rego[dat1$Groups=="P. bisulcata" & dat1$New.level=="P20"] <-"pos-survivor"

dat1$Rego[dat1$Groups=="indeterminable" & dat1$New.level=="P20"] <-"pos-survivor"

dat1$Rego[dat1$Groups=="Passaloteuthis sp. juv" & dat1$New.level=="P20"] <-"pos-survivor"

dat1$Rego[dat1$Groups=="P. cf. longiformis" & dat1$New.level=="P20"] <-"pos-survivor"

dat1$Rego[dat1$Groups=="P. milleri" & dat1$New.level=="P20"] <-"pos-survivor"

possurvivors <- subset(dat1, Rego == "pos-survivor")

possurvivorsL <- ddply(possurvivors, .(New.level), summarise, med = median(GM.1, na.rm=TRUE),freq=length(GM.1))

########calculate medians per bed for all specimens per bed (assemblage scale)

dat1 <- read.csv(file="epirostrum.csv", header=T, sep=";")

dat1<-dat1[is.na(dat1$GM.1)==F,]

dat1$Level <- ordered(dat1$New.level, levels = c("P925", "P949", "P961", "P982", "P984", "P8", "P12", "P14", "P20", "P133"))

alltaxaGM <- ddply(dat1, .(Level), summarise, med = median(GM.1, na.rm=TRUE),freq=length(GM.1))
